# Development of a complex intervention to prevent complications in patients recovering at home after transcatheter aortic valve replacement by optimizing home-based cardiac rehabilitation processes: a Delphi study

**DOI:** 10.3389/fpubh.2024.1491311

**Published:** 2024-12-18

**Authors:** Ying Ying Jia, Zhi Tin Guo, Yu Ping Zhang, Jian Ping Song

**Affiliations:** ^1^Nursing Department, The Second Affiliated Hospital of Zhejiang University School of Medicine, Hangzhou, Zhejiang Province, China; ^2^Nursing Department, Zhejiang University School of Medicine, Hangzhou, Zhejiang Province, China

**Keywords:** transcatheter aortic valve replacement, home-based, Delphi, cardiac rehabilitation, adherence

## Abstract

**Objectives:**

This study aimed to systematically develop a nurse-led complex intervention to enhance the quality of and adherence to home-based cardiac rehabilitation (HBCR) care for patients who have undergone transcatheter aortic valve replacement (TAVR). The intervention integrated stakeholder perspectives, expert insights, empirical evidence, and theoretical frameworks.

**Methods:**

We initially searched for initial cardiac rehabilitation strategies based on the “Behavior Change Wheel” model and literature review. The Delphi method was used in three rounds of consultations. The guidelines for the Conducting and Reporting of Delphi studies were also followed. The Delphi panel consisted of 15 experts in rehabilitation medicine, clinical medicine, cardiovascular nursing, behavioral science, community nursing, geriatric nursing, and nursing management from various provinces and cities in China. Panelists were asked to rate the effectiveness of HBCR strategies on a 5-point Likert scale.

**Results:**

A questionnaire was sent to the members of the expert panel via email. The consensus from 15 experts led to the development of a framework in round 3. The response rates for the three rounds were 88.23, 100.00, and 100.00%, respectively. The expert authority coefficients for all rounds were 0.95. The Kendall coefficients obtained in the three rounds were 0.09, 0.78, and 0.87, respectively.

**Conclusion:**

A set of strategies was developed for a comprehensive HBCR program for patients with TAVR, which can provide practical methods for relevant government departments, healthcare professionals, and patients’ families. Future research should investigate the experiences of stakeholders and assess the cost-effectiveness of implementing these strategies.

## Background

Aortic stenosis (AS) can be caused by conditions such as rheumatic heart disease and congenital cardiac defects and most commonly occurs in older adults (1). Transcatheter aortic valve replacement (TAVR) is the primary therapeutic intervention for AS (2, 3). Due to factors such as advanced age, frailty, and reduced left ventricular function, patients with TAVR are prone to high-degree or complete atrioventricular block, cardiac tamponade, and other complications (4). Patients with TAVR have a one-year readmission rate of 44.2% and an all-cause mortality rate of 23.7% (5–7). These patients require lifelong management (8).

Cardiac rehabilitation (CR) is a comprehensive program aimed at improving cardiac function, health status, and the quality of life of patients with cardiac diseases (9). In 2018, approximately 55% of countries and regions worldwide provided CR, but the referral process, participation, and completion rates were not satisfactory (10). High healthcare costs, poor patient adherence, and uneven distribution of healthcare resources constrain the use of outpatient CR (11).

Home-based cardiac rehabilitation (HBCR) can serve as systematic and appropriate medical services for patients with TAVR in settings other than medical institutions, such as communities, homes, and workplaces. It can also promote cardiac function recovery and reduce healthcare costs (12). Considering the aging population and coronavirus disease 2019 pandemic, the American Heart Association (AHA) has advocated for the global implementation of digital HBCR (13, 14).

Previous interventions within HBCR have encompassed several pivotal measures, including exercise interventions, nutritional support, psychological support, and pharmacological support. A systematic review reveals that HBCR predominantly focuses on exercise interventions, with significant gaps in nutritional, psychological, and pharmacological support (15, 16). Regarding safety assurance, technologies such as wearable heart rate monitors, mobile electrocardiogram systems, and telephone communications are primarily used for real-time patient data monitoring (17). But poor adherence to HBCR is common among patients with TAVR (18). Factors influencing their adherence to HBCR include individual and social support, rehabilitation environment, community management policies, and others (19). Strategies to improve adherence to HBCR include cultivating patients’ motivation for HBCR, stimulating patients’ motivation for recovery, improving the HBCR environment, enhancing patients’ self-care abilities, and reducing the economic costs associated with HBCR (20). Current studies on HBCR have reported on few individualized elements, large heterogeneity among the intervention, and uneven home rehabilitation quality levels (21–24). Moreover, information on how to effectively apply these strategies in clinical practice is limited (25). Therefore, developing effective strategies to strengthen adherence to HBCR in the era of “big data” is crucial.

We conducted Delphi expert consultations based on the theoretical framework of the transtheoretical model of the Behavior Change Wheel (BCW) to develop actionable strategies to eliminate the barriers to HBCR adherence and provide basis for HBCR for patients post-TAVR.

## Methods

### Study design

This study conforms to the standards for the conducting and reporting of Delphi studies guidelines (26). The Delphi method is suitable for interdisciplinary research on complex issues. It ensures the anonymity and objectivity of expert opinions through multiple rounds of feedback to achieve reliable consensus. This method not only enhances the quality of decision-making but also improves the operability and reliability of results through systematic feedback mechanisms.

This research aims to construct a digitalized HBCR intervention scheme by synthesizing diverse perspectives, focusing on behavior change techniques, and incorporating digital innovations. In contrast to conventional rehabilitation methods, behavior change techniques emphasize active modification and ongoing management of patient behaviors, thereby enhancing patient adherence and rehabilitation efficacy. The integration of digitally innovative technologies offers new possibilities for the dissemination and promotion of HBCR.

### Literature search

The team followed the principle of searching from top to bottom in layers according to the “6S model” and comprehensively retrieved relevant evidence. The resources searched include BMJ Best Practice, UpToDate, Clinical Key, the Agency for Healthcare Research and Quality (AHRQ), the National Institute for Health and Care Excellence (NICE), AHA website and the American Association of Cardiovascular and Pulmonary Rehabilitation (AACVPR) website, EBSCO, ACP Journal Club, Joanna Briggs Institute (JBI), the Cochrane Library, Embase, PubMed, Web of science, the China National Knowledge Infrastructure. The retrieval period covered July 1, 2013, to July 1, 2023. The search strategy combined MeSH descriptors with unrestricted search terms. The English search lexicon is delineated as follows: “transcatheter aortic valve replacement/transcatheter aortic valve implantation/TAVR/TAVI/bioprosthetic valve/heart valve prosthesis/heart valve prosthesis implantation/mechanical heart valve/bioprosthetic heart valve/valve” in conjunction with “cardiac rehabilitation/heart rehabilitation/cardiovascular rehabilitation/rehabilitation training/exercise training/home-based cardiac rehabilitation.”

The inclusion criteria for the literature search were as follows: (1) Study population: Patients diagnosed with aortic stenosis who underwent TAVR; (2) Study content: Research pertaining to HBCR interventions encompasses a comprehensive array of approaches, including exercise regimes, psychological interventions, nutritional strategies, smoking cessation initiatives, pharmacological interventions, and the synergistic application of multiple intervention modalities; (3) Language of the literature: Chinese or English; (4) Research type: Guidelines, expert consensus, evidence summaries, clinical decision-making, recommended practices, systematic reviews, randomized controlled trials, and (5) Exclusion criteria: Duplicate publications and literature without full-text access.

### Behavior change wheel

The BCW theory, which consists of a three-layer ring structure with core, intermediate, and outer layers, was proposed by Michie et al. (27) in 2011. It can be used to analyze the mechanisms and influencing factors of individual behaviors and guide the design of intervention programs (28). The core layer of the theory assumes that individual behavior requires and is influenced by three conditions: ability, opportunity, and motivation. The remaining two layers are intervention functions and policy categories (29).

The BCW believes that human behavior is not only a personal choice but a product of the integrated role of the environment. It emphasizes the interaction between the individual and social system environment and attempts to explain behavior from multiple levels such as the individual, community, and environmental levels (29, 30). Furthermore, the BCW theory emphasizes the specificity and practicality of interventions, crafting strategies tailored to the barriers and facilitators of behavior change, thereby assisting healthcare professionals in delivering effective intervention plans that enhance adherence to HBCR. Additionally, BCW can be integrated with various frameworks and techniques, such as the Theoretical Domains Framework and behavior change technologies, to deepen the inquiry into behavior modification. Thus, this study has elected to utilize the BCW theoretical framework as a guiding beacon in the orchestration of the intervention program.

### The panel of experts

The findings of the Delphi method merely represent the statistical distribution of the opinions of the expert panel. The conclusions drawn are contingent upon the scope of expertise, the sources of information, and the methods of analyzing information employed by the selected experts. This study rigorously adheres to the selection criteria based on the experts’ professional backgrounds and knowledge domains. We employed purposive sampling to select experts to participate in the Delphi expert consultations (31). The inclusion criteria for the experts were as follows: (1) possessing a minimum of a bachelor’s degree in a relevant field; (2) holding an intermediate or a senior level professional title; (3) having a minimum of 10 years of experience in nursing quality management, CR, clinical medicine, geriatric nursing, community nursing, rehabilitation medicine, and behavioral science; and (4) volunteering for the expert consultation.

We identified experts through the collaborative network of the cardiovascular nursing professional committee of the Chinese Nursing Association. Our goal was to recruit experts from different provinces and cities in China to ensure that the recommendations align with the needs of low-and middle-income families. The size of the expert group is based on the data saturation principle (32). In our study, data saturation was achieved when the data from the fifteenth expert was included.

### The development of the preliminary draft for the intervention scheme

The development of the program follows eight distinct steps (27). (1) Defining the behavioral problem in behavioral terms: The poor adherence of TAVR patients to HBCR. (2) Selecting the target behavior: Enhancing the adherence of TAVR patients to HBCR. (3) Specifying the target behavior: Elevating the adherence of TAVR patients to follow-up management, exercise management, cardiovascular risk factor management, and complication observation. (4) Identifying what needs to be changed: Numerous studies indicate that psychological factors such as anxiety and depression, the health literacy of the patients, self-efficacy, the patients’ perception of the benefits of exercise rehabilitation, motivation for exercise rehabilitation, knowledge levels regarding rehabilitation, exercise apprehension, and social support are crucial factors influencing adherence to CR (33–35). (5) Identifying intervention functions: The team determined the intervention functions based on the criteria of affordability (A), practicability (P), effectiveness and cost-effectiveness (E), acceptability (A), side-effects or safety (S), and equity (E), collectively known as the APEASE standards. (6) Identifying policy categories: The BCW framework encompasses seven policy categories, including communication/marketing, guidelines, fiscal measures, regulations, legislation, environmental/social planning, and service provision. Aligning with the APEASE criteria, this study aims to develop a HBCR program for TAVR patients, with the aspiration of codifying it into a collective standard for implementation and dissemination. (7) Identifying behavioral change techniques: Upon selecting the intervention function and policy category, the intervention designer maps the behavior change techniques to the respective intervention function, based on the APEASE criteria. (8) Identifying the implementation model: Based on the APEASE standards, the research opted to implement group rehabilitation interventions through WeChat mini-programs. This research team has independently developed an I Nursing WeChat Mini Program, which is provided free of charge for patients.

### Instrument, data collection, and analysis

The questionnaire used in this study consists of four parts: introduction, general information survey for experts, main text, and self-assessment form for experts. The first section provides an overview of the research background and objectives. The survey section incorporates research orientation, years of CR experience, educational attainment, and professional title. The main text section comprises the importance of the items, retention, and deletion of the items, as well as expert opinions on the items. The expert panel independently rated each item on a 5-point Likert scale (ranging from “very important” to “not important”) and provided suggestions for each item. The last section of the questionnaire assesses the experts’ basis for making conclusions on and familiarity with the research content.

Three rounds of Delphi surveys were conducted via email from August to October 2023, each lasting for 4 weeks. To increase the effective response rate of the questionnaires, reminder e-mails were sent 1 and 2 weeks after each questionnaire was sent. In each round of research, detailed contextual information and evidence-based medical data are meticulously provided to support the experts’ fact-based judgments. An experienced advanced-practice cardiovascular nurse collected and analyzed the data.

In the statistical summary of the collected questionnaires, criteria such as mean importance >4 and coefficient of variation (CV) > 0.3 were utilized to screen the rated items at various levels. The selected items were then subjected to discussion and analysis. Additionally, some items were added, merged, and modified according to the expert opinions. After the third round of Delphi consultation, the expert opinions became more focused and convergent. Therefore, the rehabilitation program framework for patients with TAVR was successfully developed.

### Statistical method

The original data was entered into and sorted using Excel 2016 before being transferred to SPSS 27.0. Continuous data are expressed as mean and standard deviation. Categorical data are expressed as counts, percentages, and other statistics used in the Delphi analysis (full-score ratio, CV, coefficient of coordination, and degree of expert authority). Expert participation coefficient was measured using the questionnaire response rate and treated as an indicator of active involvement. The coefficient of expert authority was calculated using the formula *C*_r_ = (*C*_a_ + *C*_s_)/2, where *C*_a_ represents the expert’s criteria for judging the indicators and *C*_s_ represents their level of familiarity with the indicators.

## Results

### Literature search results

The preliminary search yielded a total of 762 relevant articles. After deduplication, 495 unique articles were obtained. After excluding conference abstracts, guideline interpretations, and other literature types, 88 articles remained. After reading the full texts, 16 articles were ultimately included in this study.

Our research team synthesized the best evidence for HBCR from the literature. Using the best summarized evidence as a foundation and the BCW as a framework, we established a preliminary index system for HBCR in patients with TAVR.

### Characteristics of participants

A total of seven Grade III-A hospitals with 15 experts from three provinces in China, including Beijing, Zhejiang, and Gansu were finally selected for the study. The experts had a mean age of 40.13 ± 6.95 years and mean working experience of 17.60 ± 9.13 years. The detailed information on the experts is listed in Table 1.

**Table 1:** General information sheet on the inclusion of experts.

Categories	Clusters	Frequency	Component ratio (%)
Age	30–39	9	60.0%
	≥40	6	40.0%
Gender	Male	5	33.3%
	Female	10	66.6%
Educational background	Bachelor	5	33.3%
	Master	5	33.3%
	Doctor	5	33.3%
Years working in rehabilitation	<10	2	13.3%
	10–19	8	53.3%
	≥20	5	33.3%
Subject category	Clinical medicine	2	13.3%
	Cardiovascular nursing	4	26.6
	Rehabilitation science	2	13.3%
	Nursing management	3	20.0%
	Behavioral science	1	6.66%
	Community nursing	1	6.66%
	Geriatric nursing	2	13.3%
Professional title	Intermediate professional title	6	40.0%
	Title at the deputy senior level	5	33.3%
	High-level professional title	4	26.6%

### Expert enthusiasm and authority level

The positive coefficient of Delphi experts is estimated as the effective response rate of the questionnaire. These response rates for the first, second, and third rounds of consultations were 88.23, 100, and 100%, respectively (Table 2). The expert authority coefficient for all rounds of the Delphi expert consultation was 0.95, indicating a good expert authority (Table 3).

**Table 2:** Positivity of experts.

	Number of questionnaires issued	The number of questionnaires effectively returned	Effective recovery rate (%)	Number of experts making recommendations
Round 1	17	15	88.23	15
Round 2	15	15	100	4
Round 3	15	15	100	0

**Table 3:** Degree of authority of the experts.

	Familiarity coefficient (Ca)	Judgment coefficient (Cs)	Authority coefficient (Cr)
Round 1	0.92	0.97	0.95
Round 2	0.93	0.97	0.95
Round 3	0.93	0.97	0.95

### Degree of consistency in expert opinions

The degree of consensus in the expert opinions is represented by Kendall’s coefficient of concordance. A higher Kendall’s *W* value indicates a greater agreement among the experts and better coordination of the items (36). The Kendall *W* values for the three rounds were 0.09 (*p* < 0.05), 0.78 (*p* < 0.001), and 0.87 (*p* < 0.001), respectively (Table 4).

**Table 4:** The result of expert opinions’ coordination degree.

	Expert (n)	CV	Kendall’s (W)	*χ*^2^	*p*-value
Round 1	15	0–0.40	0.09	111.534	*P* < 0.05
Round 2	15	0–0.40	0.78	511.204	*P* < 0.001
Round 3	15	0–0.29	0.87	869.014	*P* < 0.001

### Degree of consistency in expert opinions

Finally, the basic structure of the proposed program for the CR of patients with TAVR was determined by 7 primary indicators, 18 secondary indicators, and 45 third-level indicators (Table 5).

**Table 5:** A comprehensive intervention strategy for HBCR in patients with TAVR.

First-level indicator	Second-level indicator	Third-level indicator
A. Risk assessment for HBCR in patients with TAVR	A1. Formation of a multidisciplinary team for HBCR in patients with TAVR	A1.1 Select staff with 3 or more years of relevant work experience to form a team with a clear staff-to-patient ratio and an appropriate hierarchical composition within the team.
		A1.2 Develop a training and assessment program for home rehabilitation teams for patients with TAVR.
		A1.3 Develop an emergency procedure for telemedicine HBCR.
	A2. Screening patients and conducting individualized risk assessments	A2.1 Develop exclusion criteria for HBCR to exclude patients with conditions such as unstable angina and severe arrhythmias.
		A2.2 The first individualized assessment includes the patient’s medical history, laboratory findings, debilitation, nutrition, motor and cognitive function, valvular function, associated complications and comorbidities, flexibility and balance, activities of daily living, quality of life, psychology, digital health literacy, household economic circumstances, and the presence of deep vein thrombosis.
		A2.3 Patient health records are established with comprehensive information on basic patient information and disease history.
		A2.4 Dynamic assessment: patients are reminded weekly to fill out tools such as medication adherence questionnaires and exercise adherence scales to assess their adherence. The team learns about the patient’s recovery and feelings through inspirational short message service and adjusts the rehabilitation program in time according to the relevant indicators.
B. Fostering motivation for HBCR in patients with TAVR	B1. Fostering reflective motivation for HBCR in patients with TAVR	B1.1 In conjunction with in-hospital health promotion, the platform publishes a weekly video on the philosophy, importance, benefits, and health outcomes of HBCR.
		B1.2 In conjunction with in-hospital health education, the platform publishes the incidence, influencing factors, and harmful effect of valvular disease every week to highlight the susceptibility of patients and their caregivers to adverse outcomes.
	B2. Fostering spontaneous motivation for HBCR in patients with TAVR	B2.1 Individualized rehabilitation programs are developed in conjunction with risk assessment results, patient preferences, and patient punch cards, which are recorded through an app.
		B2.2 Recognize patients based on their cumulative monthly points (award virtual medals or other rewards).
C. Home risk factor management in patients with TAVR	C1. Provide knowledge about risk factor management after TAVR	C1.1 In conjunction with in-hospital health promotion, the platform publishes weekly videos on risk assessment for patients and their caregivers, explaining the content, purpose, and importance of risk assessment.
		C1.2 In conjunction with in-hospital health education, the platform publishes weekly videos for patients and their caregivers on postoperative risk health education regarding heart valve disease, mental health, and sleep management.
		C1.3 In conjunction with the hospital’s health education mission, the platform publishes postoperative medication videos once a week, explaining the use of commonly used antithrombotic drugs.
	C2. Provide skill instructions for post-TAVR risk factor management	C2.1 Combining face-to-face teaching in the hospital with remote video teaching, the purpose for and method of using the platform and wearable devices were explained.
		C2.2 Combining face-to-face teaching in the hospital with remote video teaching, patients are taught to create, view, and manage rehabilitation programs and goals.
		C2.3 Combining face-to-face teaching in the hospital with remote video teaching, patients and their caregivers are taught self-monitoring techniques (monitoring of blood pressure, weight, etc.), recognition of dangerous symptoms, and first aid skills.
		C2.4 Combining face-to-face teaching in the hospital with remote video teaching, patients and their caregivers are equipped with the use of portable coagulometers.
	C3. Improving adherence to home-based risk factor management in patients with TAVR	C3.1 Based on the patient’s condition, set management goals for blood pressure, lipids, blood glucose, smoking cessation, and other risk factors, and the patient or his/her caregiver uses the platform to upload the patient’s health data (once a week). For those who do not know how to use smart devices, telephone follow-up visits are conducted.
		C3.2 The platform sends information such as healthy diet to the patient and his/her caregiver at regular intervals throughout the day to remind the patient to implement rehabilitation measures. If there are abnormalities in the patient’s data, the platform sends alerts to the rehabilitation team and the patient’s caregivers about abnormal monitoring values.
		C3.3 If patient compliance is good, daily reminders are changed to once-a-week reminders, and weekly reminders are changed to once-a-month reminders. If patient compliance is bad, they were sent at the original frequency.
D. Symptom and follow-up management of patients with TAVR during home rehabilitation	D1. Provide knowledge on symptom management and follow-up after TAVR surgery	D1.1 In conjunction with in-hospital education, the platform publishes weekly videos on prevention, symptoms, and management measures for postoperative long-term complications.
		D1.2 In conjunction with in-hospital education, the platform publishes weekly videos on home follow-up visits, including the purpose, timing, importance, and contents of the checkups, and precautions for follow-up visits.
	D2. Develop a home follow-up program for patients with TAVR	D2.1 Follow-up visits were conducted 1, 3, 6, and 12 months after discharge, and follow-up reminder messages were sent to patients 3 days before and on day of the follow-up visit.
		D2.2 A follow-up message reminder is sent to the rehabilitation team via the platform 2 days before the follow-up visit, confirming the patient’s participation in the follow-up visit and booking screening and laboratory tests for the patients in advance.
E. Home exercise management in patients with TAVR	E1. Provide knowledge about home exercise for patients with TAVR	E1.1 In conjunction with in-hospital face-to-face teaching, the platform publishes home exercise videos for patients and their caregivers. The content includes the type, intensity, frequency, and duration of exercise; adverse reactions; and countermeasures during exercise.
	E2. Provide skill instructions for home exercises after TAVR	E2.1 Combining in-hospital face-to-face instruction with remote video instructions, the program provides patients and their caregivers with skills instruction in home-based aerobic endurance exercise, standing balance and motor coordination training, resistance training exercise, and Chinese medicine rehabilitation exercise.
	E3. Continuous improvement of physical strength and endurance in patients with TAVR	E3.1 The platform sends exercise reminders to patients on Mondays, Wednesdays, and Fridays each week.
		E3.2 Encourage patients and their caregivers to use pedometers, portable electrocardiographs, and health diaries to monitor exercise dynamically.
F. Creating a home recovery environment for patients with TAVR	F1. Provision of HBCR materials and equipment and strengthening of HBCR safety monitoring	F1.1 Before a patient is discharged from the hospital, the rehabilitation team provides the patient with equipment, such as wearable devices, to dynamically monitor exercise metrics and ensure the patient’s exercise safety.
		F1.2 The team creates health promotion materials and distributes them to patients before discharge.
		F1.3 The rehabilitation team establishes an HBCR system that combines smart wearable devices, mobile apps, and a systematic website on healthcare to dynamically monitor patients’ recovery.
	F2. Strengthening social integration and rebuilding the social environment for the rehabilitation of patients with TAVR	F2.1 Patients and their caregivers are encouraged to join rehabilitation groups and actively participate in activities such as cardiac rehabilitation workshops and in-hospital communication salons.
		F2.2 Personal tips are shared by patients and caregiver with good compliance. In addition, the rehabilitation team selects the best patient and caregiver every quarter and awards them with e-certificates.
		F2.3 The rehabilitation team helps patients identify and overcome barriers that affect health-related behaviors and provides targeted assistance to patients who live alone and have low education levels.
	F3. Enhancing family interventions to rebuild the HBCR environment for patients with TAVR	F3.1 The rehabilitation team has established an at-home cardiac rehabilitation system that integrates smart wearable devices, mobile applications, and the healthcare provider’s system website to dynamically monitor the patient’s recovery progress.
	F4. Building the telemedicine environment of the rehabilitation team	F4.1 Create a suitable space and environment for teleconsultations to ensure that the team is not disturbed during telemedicine consultations.
		F4.2 Hospital establishes an online cardiovascular nurse clinic and cardiac rehabilitation clinic.
G. Policies and regulations related to digital HBCR	G1. Robust TAVR telemedicine quality management system	G1.1 System for handling adverse incidents and disputes.
		G1.2 Establishment of an exit mechanism and benefit guarantee system for rehabilitation team members.
		G1.3 Development of a patient privacy protection system.
		G1.4 Development of a patient safety and protection system.
		G1.5 Development of a system for recording telemedicine instruments.
	G2. Remote charging regulations for rehabilitation after TAVR	G2.1 TAVR rehabilitation telemedicine fees are clear and reasonable.
		G2.2 Development of an incentive program for remote rehabilitation of patients with TAVR (virtual prizes).

### Strategies to support HBCR adherence for patients with TAVR

#### First-level indicators

Among the first-level indicators, the experts recommended the addition of “Risk assessment for HBCR in patients with TAVR.” They agreed that the first HBCR risk assessment should be performed before the patient is discharged from the hospital. A dynamic HBCR risk assessment should be conducted while the patient is at home.

### Second-level indicators

Experts proposed and added two second-level indicators (“A1. Formation of a multidisciplinary team for home rehabilitation of patients with TAVR” and “A2. Screening of patients and conducting of individualized risk assessments”) under the first-level indicators (A. Risk assessment for HBCR in patients with TAVR). Motivation is defined as the function that directs and energizes behavior based on the content and magnitude of the target. Motivational strategies are defined as specific strategies, techniques, or methods that guide patient rehabilitation (37). Experts suggested that patients’ motivation for rehabilitation should be fostered before managing CR risk factors such as exercise and nutrition. Thus, the first-level entry (B. Fostering motivation for HBCR) was moved to the back of the HBCR risk assessment indicator.

#### Third-level indicators

##### Methods and targets of health promotion

The experts suggested that all health education should be provided by combining in-hospital and out-of-hospital health education. Health education should also consider that patients with TAVR are mostly older adults with advanced age and co-morbidities who have limited comprehension. Health education should be provided to patients and their primary caregivers.

##### Funding management for digital HBCR

Experts suggested that rehabilitation teams should equip patients with wearable devices and assess their home conditions. Regarding the reward mechanism for CR, experts believe that the reward of unlocking new props does not apply to patients with TAVR who are mainly older adults. Therefore, we modified the rewards to virtual medals and certificates of honor. The experts believed that establishing a community environment for the rehabilitation of patients with TAVR was more difficult if the community service center had only one patient with TAVR. Therefore, the phrase “organizing community rehabilitation exchange salons” was revised to “organizing in-hospital rehabilitation exchange salons.”

##### Capacity for self-care

In the section on cardiovascular risk factor management, “Encourage patients to use the platform to upload data such as waist circumference, sleep, and weight, and provide regular feedback to patients” did not specify how often patients should record these data. Therefore, the research team revised this section to “Patients should record their weight and waist circumference once a week.”

##### Combining online and offline strategies to organize rehabilitation activities

“The rehabilitation team regularly selects the best patient and caregiver and awards e-certificates to increase patient and caregiver motivation.” This activity is performed online, so its implementation may not be ideal. The experts recommended that this content should be placed in the section on rebuilding the social environment. Therefore, the original statement was modified to “Organize an offline salon in the hospital, select patients and caregivers with better compliance, and award them with certificates while.” Considering that offline communication enhances clear communication and the understanding of the needs of patients, “Develop a personalized rehabilitation program through online communication, considering the patient’s preferences, and records the patient’s punch cards through the app to motivate the patient’s execution of CR” was revised to “Conduct a patient needs assessment before discharge, and assist in developing a personalized HBCR program that considers the patient’s preferences.”

## Discussion

In our study, we drew on the knowledge of experts experienced in care management, behavioral sciences, and community care to develop a set of strategies to improve adherence to HBCR of patients with TAVR. Researchers can employ various metrics during the implementation of the program, such as the six-minute walking distance, the activities of daily living scale, incidence of post-TAVR complications, readmission rates, and mortality rates. They can also gather data through patient satisfaction surveys, economic benefit analyses, long-term follow-ups, and tracking studies. By continuously refining the intervention program based on these metrics and studies, to expand the coverage of cardiac rehabilitation.

Adherence to HBCR is critical for improving patient prognostic outcomes. Developing rehabilitation strategies may have a significant impact on the long-term success of HBCR (38). TAVR across hospitals in China is characterized by a high proportion of procedure volume in large medical centers and varying levels of overall technological proficiency among hospitals. Therefore, this study mainly recruited relevant experts from cities with a higher volume of TAVR. The included experts have extensive experience in areas such as rehabilitation medicine, cardiovascular rehabilitation, clinical medicine, and geriatric nursing. The selection of experts is comprehensive and representative, ensuring that the developed CR strategies meet the needs of HBCR for patients with TAVR. The authority coefficient *C*_r_ is greater than 0.9, indicating that the degree of expert authority is high. In addition, Kendall’s *W* of the third round of expert consultation is 0.87 (*p* < 0.001), indicating that the expert’s opinion tends to be consistent.

The intervention scheme is divided into seven distinct domains, a categorization that aligns seamlessly with the findings of existing systematic reviews (39, 40). Both studies underscore the importance of promoting tele-education and monitoring based on digital healthcare, consistent with the findings (41, 42). Intervention studies show that adherence can be enhanced through comprehensive health education, exercise monitoring, providing feedback, setting goals, and applying behavior modification techniques among patients” (43, 44). In contrast, this study, which is based on integrating behavioral modification techniques, motion monitoring, and various other technologies into the intervention framework through the Delphi consultative process, can effectively enhance adherence.

It is important to give patients with TAVR and their caregivers the necessary support and guidance (45). In this study, the intervention protocol was geared toward patients and their caregivers. Considering that patients with TAVR are mostly older adult and co-morbid, and may have difficulty using technology such as mobile devices (46). In terms of teaching methods, face-to-face teaching pays more attention to the physiological and psychological changes of the subjects. The intervention team can provide clear and user-friendly technical instructions and support services during face-to-face teaching sessions, thereby reducing complexity in usage. Additionally, they can incorporate interactive elements such as demonstration exercises and practical drills, complemented by supplementary tools like guides and video tutorials for enhanced instruction (46). In comparison, web-based teaching can break the limitations of time and space so that the older adult have more opportunities to practice independently (46). Accordingly, the program developed by the research team fully combines the advantages of these two teaching methods and, thus, is more applicable. It should be noted that although web-based courses are becoming more common, researchers still need to consider some barriers that present to older adults during program implementation. The research team may impart comprehensive technological training to patients and their caregivers to ensure rapid proficiency. Moreover, dedicated support staff could be designated to assist patients and their caregivers throughout the intervention, particularly during the initial stages of recovery. Additionally, a robust ongoing technical support system could be established, enabling patients and their caregivers to seek assistance at any time as needed, interventionists can gather feedback through a variety of channels. This feedback should then be meticulously analyzed and utilized to refine and optimize the intervention strategies (47).

**Figure 1:**
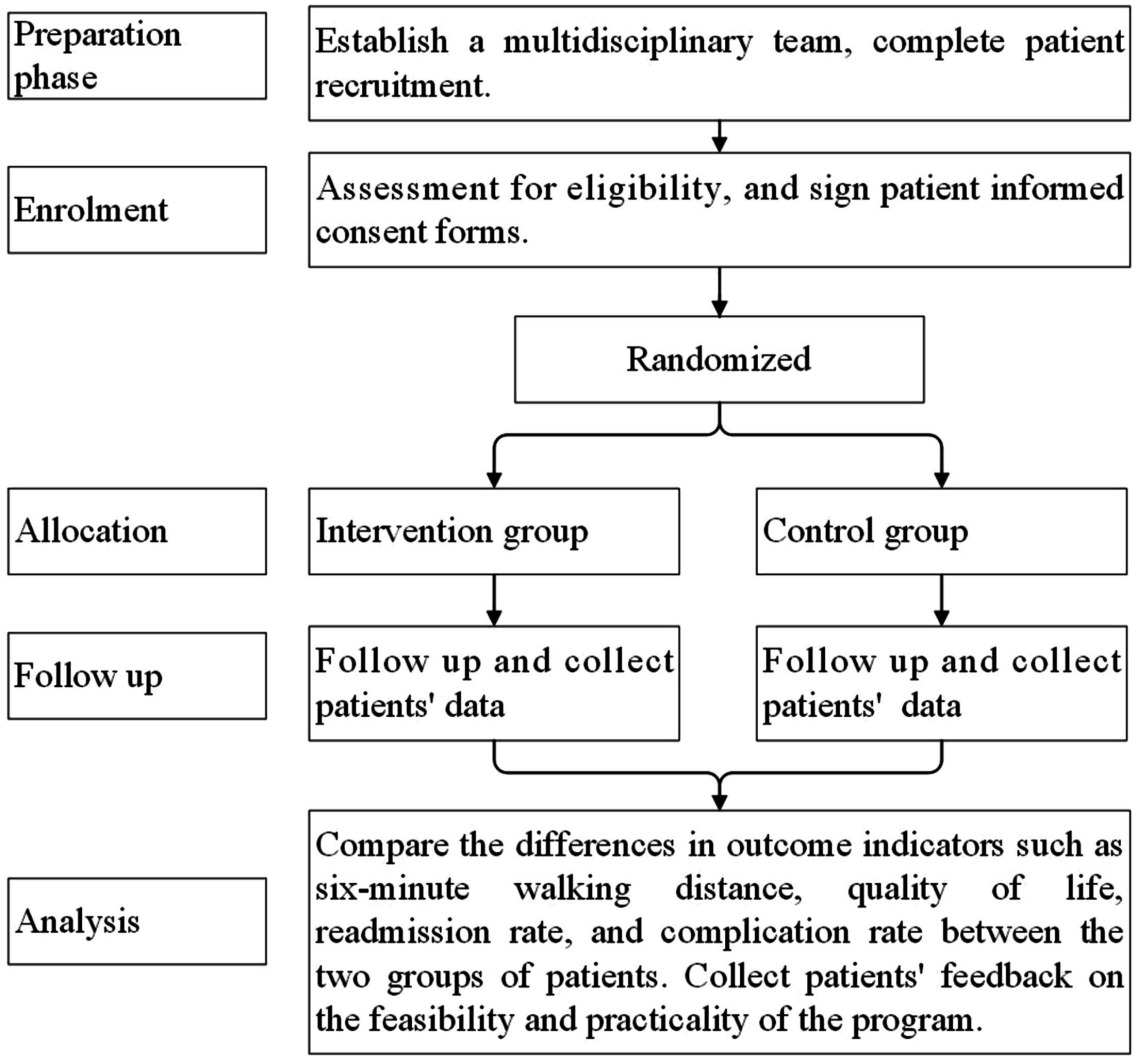
Flowchart of pilot study of intervention plan.

Prior to the widespread implementation and dissemination of the proposed scheme, pilot studies and cost-effectiveness analyses must be conducted. As illustrated in Figure 1, the flowchart offers a lucid framework for the pilot research of the scheme. During the pilot research phase, the research team can employ the HBCR compliance questionnaire developed by Yang et al. (48) to assess patients’ adherence. When significant improvements in patients’ health metrics are observed alongside high levels of patient compliance and satisfaction, consideration can be given to initiating formal studies on the scheme’s application. Studies indicate that, compared to traditional care, the HBCR scheme demonstrates superior cost-effectiveness in treating patients with heart failure (49). Throughout the scheme’s application, scholars can construct Markov models to conduct precise cost-effectiveness analyses, thereby laying a robust foundation for its large-scale dissemination.

The strength of this study lies in the interdisciplinary nature of the recruited panel of experts. This ensures that the developed HBCR strategies are scientifically sound and reliable. Furthermore, based on the transtheoretical model of BCW, this study makes a significant contribution toward improving patients’ adherence to HBCR. However, there are still some limitations in this study. For instance, we did not include TAVR patients and their families in the Delphi panel. Besides, due to time constraints, the newly developed CR program in this study has not undergone clinical application research, and the applicability and sensitivity of the CR program need further investigation.

## Conclusion

In this study, we constructed an intervention framework for HBCR for patients with TAVR based on CBM using a modified Delphi study. The strategies were categorized into seven domains: risk assessment for home, fostering motivation for HBCR for patients with TAVR, home risk factor management in patients with TAVR, symptom and follow-up management of patients with TAVR, home exercise management in patients with TAVR, creating a home recovery environment for patients with TAVR, policies and regulations related to digital HBCR. The method of constructing the intervention program is scientific and reliable, and the content is specialized. Healthcare professionals and patients’ families can be guided by the program to carry out high-quality HBCR for patients with TAVR to improve the prognostic outcomes of patients.

## Data Availability

The raw data supporting the conclusions of this article will be made available by the authors, without undue reservation.

## References

[1] Ribeiro GS Melo RD Deresz LF Dal Lago P Pontes MR Karsten M. Cardiac rehabilitation programme after transcatheter aortic valve implantation versus surgical aortic valve replacement: systematic review and meta-analysis. Eur J Prev Cardiol. (2017) 24:688–97. doi: 10.1177/2047487316686442, PMID: 28071146

[2] Howard C Jullian L Joshi M Noshirwani A Bashir M Harky A. TAVI and the future of aortic valve replacement. J Card Surg. (2019) 34:1577–90. doi: 10.1111/jocs.14226 31600005

[3] Webb JG Blanke P Meier D Sathananthan J Lauck S Chatfield AG. TAVI in 2022: remaining issues and future direction. Arch Cardiovasc Dis. (2022) 115:235–42. doi: 10.1016/j.acvd.2022.04.001, PMID: 35527211

[4] Otto CM Kumbhani DJ Alexander KP Calhoon JH Desai MY Kaul S. 2017 ACC expert consensus decision pathway for Transcatheter aortic valve replacement in the Management of Adults with Aortic Stenosis: a report of the American College of Cardiology Task Force on clinical expert consensus documents. J Am Coll Cardiol. (2017) 69:1313–46. doi: 10.1016/j.jacc.2016.12.006, PMID: 28063810

[5] Elbaz-Greener G Qiu F Webb JG Henning KA Ko DT Czarnecki A. Profiling hospital performance on the basis of readmission after Transcatheter aortic valve replacement in Ontario, Canada. J Am Heart Assoc. (2019) 8:e012355. doi: 10.1161/jaha.119.012355, PMID: 31165666 PMC6645639

[6] Holmes DR Jr Brennan JM Rumsfeld JS Dai D O'Brien SM Vemulapalli S. Clinical outcomes at 1 year following transcatheter aortic valve replacement. JAMA. (2015) 313:1019–28. doi: 10.1001/jama.2015.1474 25756438

[7] Zahid S Agrawal A Salman F Khan MZ Ullah W Teebi A. Development and validation of a machine learning risk-prediction model for 30-day readmission for heart failure following Transcatheter aortic valve replacement (TAVR-HF score). Curr Probl Cardiol. (2024) 49:102143. doi: 10.1016/j.cpcardiol.2023.102143, PMID: 37863456

[8] Jubran A Patel RV Sathananthan J Wijeysundera HC. Lifetime Management of Patients with Severe Aortic Stenosis in the era of Transcatheter aortic valve replacement. Can J Cardiol. (2023) 40:210–7. doi: 10.1016/j.cjca.2023.09.010, PMID: 37716642

[9] Jia YY Song JP Yang L. Can virtual reality have effects on cardiac rehabilitation? An overview of systematic reviews. Curr Probl Cardiol. (2023) 49:102231. doi: 10.1016/j.cpcardiol.2023.102231, PMID: 38052348

[10] Beatty AL Truong M Schopfer DW Shen H Bachmann JM Whooley MA. Geographic variation in cardiac rehabilitation participation in Medicare and veterans affairs populations: opportunity for improvement. Circulation. (2018) 137:1899–908. doi: 10.1161/circulationaha.117.029471, PMID: 29305529 PMC5930133

[11] Taylor RS Dalal HM Zwisler A-D. Cardiac rehabilitation for heart failure: ‘Cinderella’ or evidence-based pillar of care? Eur Heart J. (2023) 44:1511–8. doi: 10.1093/eurheartj/ehad118, PMID: 36905176 PMC10149531

[12] Lindman BR Gillam LD Coylewright M Welt FGP Elmariah S Smith SA. Effect of a pragmatic home-based mobile health exercise intervention after transcatheter aortic valve replacement: a randomized pilot trial. Eur Heart J Digit Health. (2021) 2:90–103. doi: 10.1093/ehjdh/ztab007, PMID: 34048509 PMC8139414

[13] Thomas RJ Beatty AL Beckie TM Brewer LC Brown TM Forman DE. Home-based cardiac rehabilitation: a scientific statement from the American Association of Cardiovascular and Pulmonary Rehabilitation, the American Heart Association, and the American College of Cardiology. Circulation. (2019) 140:E69–89. doi: 10.1161/cir.0000000000000663, PMID: 31082266

[14] Golbus JR Lopez-Jimenez F Barac A Cornwell WK Dunn P Forman DE. Digital Technologies in Cardiac Rehabilitation: a science advisory from the American Heart Association. Circulation. (2023) 148:95–107. doi: 10.1161/cir.0000000000001150, PMID: 37272365

[15] Abraham LN Sibilitz KL Berg SK Tang LH Risom SS Lindschou J. Exercise-based cardiac rehabilitation for adults after heart valve surgery. Cochrane Database Syst Rev. (2021) 2021:Cd010876. doi: 10.1002/14651858.CD010876.pub3, PMID: 33962483 PMC8105032

[16] Oz A Tsoumas I Lampropoulos K Xanthos T Karpettas N Papadopoulos D. Cardiac rehabilitation after TAVI-A systematic review and Meta-analysis. Curr Probl Cardiol. (2023) 48:101531. doi: 10.1016/j.cpcardiol.2022.101531, PMID: 36493915

[17] Stefanakis M Batalik L Antoniou V Pepera G. Safety of home-based cardiac rehabilitation: a systematic review. Heart Lung. (2022) 55:117–26. doi: 10.1016/j.hrtlng.2022.04.016, PMID: 35533492

[18] Silva BV Aguiar Ricardo I Alves Da Silva P Rodrigues T Cunha N Couto Pereira S. Home-based cardiac rehabilitation during COVID-19 pandemic: effectiveness of an educational intervention. Eur. J Prev Cardiol. (2021) 28:356. doi: 10.1093/eurjpc/zwab061.356

[19] Grace SL Turk-Adawi KI Contractor A Atrey A Campbell NRC Derman W. Cardiac rehabilitation delivery model for low-resource settings: an International Council of Cardiovascular Prevention and Rehabilitation Consensus Statement. Prog Cardiovasc Dis. (2016) 59:303–22. doi: 10.1016/j.pcad.2016.08.004, PMID: 27542575

[20] Moulson N Bewick D Selway T Harris J Suskin N Oh P. Cardiac rehabilitation during the COVID-19 era: guidance on implementing virtual care. Can J Cardiol. (2020) 36:1317–21. doi: 10.1016/j.cjca.2020.06.006, PMID: 32553606 PMC7293761

[21] Kuan PX Chan WK Fern Ying DK Rahman MAA Peariasamy KM Lai NM. Efficacy of telemedicine for the management of cardiovascular disease: a systematic review and meta-analysis. Lancet Digit Health. (2022) 4:e676–91. doi: 10.1016/s2589-7500(22)00124-8, PMID: 36028290 PMC9398212

[22] Akinosun AS Polson R Diaz-Skeete Y De Kock JH Carragher L Leslie S. Digital technology interventions for risk factor modification in patients with cardiovascular disease: systematic review and Meta-analysis. JMIR Mhealth Uhealth. (2021) 9:e21061. doi: 10.2196/21061 33656444 PMC7970167

[23] Ramachandran HJ Jiang Y Tam WWS Yeo TJ Wang W. Effectiveness of home-based cardiac telerehabilitation as an alternative to phase 2 cardiac rehabilitation of coronary heart disease: a systematic review and meta-analysis. Eur J Prev Cardiol. (2022) 29:1017–43. doi: 10.1093/eurjpc/zwab106, PMID: 34254118 PMC8344786

[24] Kenny E Coyne R McEvoy JW McSharry J Taylor RS Byrne M. Behaviour change techniques and intervention characteristics in digital cardiac rehabilitation: a systematic review and meta-analysis of randomised controlled trials. Health Psychol Rev. (2023) 18:189–228. doi: 10.1080/17437199.2023.2185653 36892523

[25] Beatty AL Beckie TM Dodson J Goldstein CM Hughes JW Kraus WE. A new era in cardiac rehabilitation delivery: research gaps, questions, strategies, and priorities. Circulation. (2023) 147:254–66. doi: 10.1161/circulationaha.122.061046, PMID: 36649394 PMC9988237

[26] Jünger S Payne SA Brine J Radbruch L Brearley SG. Guidance on conducting and REporting DElphi studies (CREDES) in palliative care: recommendations based on a methodological systematic review. Palliat Med. (2017) 31:684–706. doi: 10.1177/0269216317690685 28190381

[27] Michie S van Stralen MM West R. The behaviour change wheel: a new method for characterising and designing behaviour change interventions. Implement Sci. (2011) 6:42. doi: 10.1186/1748-5908-6-42, PMID: 21513547 PMC3096582

[28] Faija CL Gellatly J Barkham M Lovell K Rushton K Welsh C. Enhancing the behaviour change wheel with synthesis, stakeholder involvement and decision-making: a case example using the ‘enhancing the quality of psychological interventions delivered by telephone’(EQUITy) research programme. Implement Sci. (2021) 16:1–11. doi: 10.1186/s13012-021-01122-2 33990207 PMC8120925

[29] Erzse A Karim SA Rwafa-Ponela T Kruger P Hofman KJ Foley L. Identifying priority interventions using the behaviour change wheel to improve public primary school food environments in urban South Africa. Lancet Glob Health. (2023) 11:S19. doi: 10.1016/s2214-109x(23)00102-x 36866476

[30] Flint KM Fairclough DL Spertus JA Bekelman DB. Does heart failure-specific health status identify patients with bothersome symptoms, depression, anxiety, and/or poorer spiritual well-being? Eur Heart J Qual Care Clin Outcomes. (2019) 5:233–41. doi: 10.1093/ehjqcco/qcy061 30649237 PMC6613596

[31] Belton I MacDonald A Wright G Hamlin I. Improving the practical application of the Delphi method in group-based judgment: a six-step prescription for a well-founded and defensible process. Technol Forecast Soc Chang. (2019) 147:72–82. doi: 10.1016/j.techfore.2019.07.002

[32] Trotter RT. Qualitative research sample design and sample size: resolving and unresolved issues and inferential imperatives. Prev Med. (2012) 55:398–400. doi: 10.1016/j.ypmed.2012.07.003, PMID: 22800684

[33] Brito J Aguiar-Ricardo I Alves Da Silva P Valente Da Silva B Cunha N Couto Pereira S. Home-based cardiac rehabilitation – the real barriers of programs at distance. Eur J Prev Cardiol. (2021) 28:336. doi: 10.1093/eurjpc/zwab061.336

[34] Daw P Wood GER Harrison A Doherty PJ Veldhuijzen van Zanten J Dalal HM. Barriers and facilitators to implementation of a home-based cardiac rehabilitation programme for patients with heart failure in the NHS: a mixed-methods study. BMJ Open. (2022) 12:e060221. doi: 10.1136/bmjopen-2021-060221, PMID: 35831041 PMC9280226

[35] Okwose NC O'Brien N Charman S Cassidy S Brodie D Bailey K. Overcoming barriers to engagement and adherence to a home-based physical activity intervention for patients with heart failure: a qualitative focus group study. BMJ Open. (2020) 10:e036382. doi: 10.1136/bmjopen-2019-036382, PMID: 32958484 PMC7507843

[36] Shen L Yang J Jin X Hou L Shang S Zhang Y. Based on Delphi method and analytic hierarchy process to construct the evaluation index system of nursing simulation teaching quality. Nurse Educ Today. (2019) 79:67–73. doi: 10.1016/j.nedt.2018.09.021 31103843

[37] Chen Y Abel KT Janecek JT Chen Y Zheng K Cramer SC. Home-based technologies for stroke rehabilitation: a systematic review. Int J Med Inform. (2019) 123:11–22. doi: 10.1016/j.ijmedinf.2018.12.001, PMID: 30654899 PMC6814146

[38] Mentias A Keshvani N Desai MY Kumbhani DJ Sarrazin MV Gao Y. Risk-adjusted, 30-day home time after Transcatheter aortic valve replacement as a hospital-level performance metric. J Am Coll Cardiol. (2022) 79:132–44. doi: 10.1016/j.jacc.2021.10.038, PMID: 35027108 PMC10535368

[39] Popovici M Ursoniu S Feier H Mocan M Tomulescu OMG Kundnani NR. Benefits of using smartphones and other digital methods in achieving better cardiac rehabilitation goals: a systematic review and Meta-analysis. Med Sci Monit. (2023) 29:e939132. doi: 10.12659/msm.939132, PMID: 37143317 PMC10167866

[40] Cordeiro ALL da Silva MA de Almeida HM Santos P. Quality of life in patients with heart failure assisted by telerehabilitation: a systematic review and Meta-analysis. Int J Telerehabil. (2022) 14:e6456. doi: 10.5195/ijt.2022.6456, PMID: 35734389 PMC9186845

[41] Nagatomi Y Ide T Higuchi T Nezu T Fujino T Tohyama T. Home-based cardiac rehabilitation using information and communication technology for heart failure patients with frailty. ESC Heart Fail. (2022) 9:2407–18. doi: 10.1002/ehf2.13934, PMID: 35534907 PMC9288767

[42] Duan Y Li X Guo L Liang W Shang B Lippke S. A WeChat Mini program-based intervention for physical activity, fruit and vegetable consumption among Chinese cardiovascular patients in home-based rehabilitation: a study protocol. Front Public Health. (2022) 10:739100. doi: 10.3389/fpubh.2022.739100, PMID: 35392478 PMC8980353

[43] Radhakrishnan K Baranowski T O'Hair M Fournier CA Spranger CB Kim MT. Personalizing sensor-controlled digital gaming to self-management needs of older adults with heart failure: a qualitative study. Games Health Journal. (2020) 9:304–10. doi: 10.1089/g4h.2019.0222 PMC7457967 32155355

[44] Sawa R Saitoh M Morisawa T Takahashi T Morimoto Y Kagiyama N. The potential application of commercially available active video games to cardiac rehabilitation: scoping review. JMIR Serious Games. (2022) 10:e31974. doi: 10.2196/31974, PMID: 35302503 PMC8976248

[45] Chung M Butala NM Faridi KF Almarzooq ZI Liu D Xu J. Days at home after transcatheter or surgical aortic valve replacement in high-risk patients. Am Heart J. (2022) 255:125–36. doi: 10.1016/j.ahj.2022.10.080 36309128

[46] Masterson Creber R Dodson JA Bidwell J Breathett K Lyles C Harmon Still C. Telehealth and health equity in older adults with heart failure: a scientific statement from the American Heart Association. Circ Cardiovasc Qual Outcomes. (2023) 16:e000123. doi: 10.1161/hcq.0000000000000123, PMID: 37909212 PMC12083189

[47] Kruse CS Molina-Nava A Kapoor Y Anerobi C Maddukuri H. Analyzing the effect of telemedicine on domains of quality through facilitators and barriers to adoption: systematic review. J Med Internet Res. (2023) 25:e43601. doi: 10.2196/43601, PMID: 36602844 PMC9893735

[48] Yang Z Jia H Zhang F Huang H Hao X Wang A. A behavioural driving model of adherence to home-based cardiac rehabilitation exercise among patients with chronic heart failure: a mixed-methods study. J Clin Nurs. (2024) 33:531–42. doi: 10.1111/jocn.16901, PMID: 37881110

[49] Taylor RS Sadler S Dalal HM Warren FC Jolly K Davis RC. The cost effectiveness of REACH-HF and home-based cardiac rehabilitation compared with the usual medical care for heart failure with reduced ejection fraction: a decision model-based analysis. Eur J Prev Cardiol. (2019) 26:1252–61. doi: 10.1177/2047487319833507, PMID: 30884975 PMC6628466

