# Supplementary material for: Development of a complex intervention to prevent complications in patients recovering at home after transcatheter aortic valve replacement by optimizing home-based cardiac rehabilitation processes: a Delphi study
Source: Front Public Health. 2024 Dec 18;12:1491311. doi: 10.3389/fpubh.2024.1491311 (PMC11688391; doi:10.3389/fpubh.2024.1491311)
Supplement: Supplementary file 1 [file Table_1.docx]

Supplementary Material

**《****Development of a complex intervention to prevent complications in patients undergoing transcatheter aortic valve replacement recovering at home by optimizing home-based cardiac rehabilitation processes: A Delphi study》****: Expert consultation form（first round）**

Dear expert,

Hello! We are a research team from Zhejiang University School of Medicine, currently conducting a study titled ***"Development of a complex Intervention to prevent complications in patients undergoing transcatheter aortic valve replacement recovering at home by optimizing home-based cardiac rehabilitation processes: A Delphi study".*** Given your extensive professional experience and profound expertise, we cordially invite you to serve as a consulting expert for this study. We appreciate your willingness to take the time out of your busy schedule to participate in this consultation.

In China, there are approximately 25 million patients with heart valve disease. Transcatheter Aortic Valve Replacement (TAVR) is the primary treatment for patients with aortic valve stenosis, but post-patients with TAVR, due to advanced age, frailty, and various comorbidities, may experience adverse events such as infective endocarditis. Cardiac Rehabilitation (CR) can improve cardiac function and quality of life for heart disease patients. In 2018, about 55% of countries globally offered CR programs, but referral rates, participation rates, and completion rates were all suboptimal. To enhance patient compliance and address underutilization of Phase II CR, this study summarizes the best evidence for home-based cardiac rehabilitation for patients with TAVR. It utilizes the Behavior Change Wheel (BCW) framework and draws upon standards like the development of principles for health-related information on social media and the development of telehealth principles and guidelines for older adults to construct a home-based cardiac rehabilitation program for patients undergoing transcatheter aortic valve replacement.

This consultation aims to refine the framework and content of the plan to ensure its scientific and effective nature and to evaluate the importance of each item. As a member of the expert consulting group, your analysis and evaluation of the relevant content are sought. The consultation consists of two rounds. This is the first round. Your personal opinions and information as an expert will be strictly confidential. We sincerely look forward to your valuable opinions and suggestions! The questionnaire includes three parts: the first part is the basic information of the experts; the second part introduces the Theory of Planned Behavior Change; the third part is the Expert Consultation Form on the Importance of Content and Format in Constructing a Home Cardiac Rehabilitation Program for Patients Undergoing Transcatheter Aortic Valve Replacement (TAVR); and the fourth part is an investigation into the authority of the experts.

To ensure the effectiveness and timeliness of the research, please promptly return the completed consultation form to the email address: 22218917@zju.edu.cn. Once again, thank you sincerely for your strong support of this research!

Contact Name: YINGYING JIA

Contact Phone: 1829843725

**Part One: Experts’ Profile**

| Name |  | Age |  | Occupation |  | Job titles |  |
| --- | --- | --- | --- | --- | --- | --- | --- |
| Highest level of education |  | Years working in a hospital |  | Contact Phone |  | Email |  |
| Hospital names |  | | | | | Experts’ department |  |
| Study field (multi-optional): | □Clinical nursing □Nursing management □Evidence-based nursing □Clinical medicine □Medical management □Cardiovascular nursing □Rehabilitation medicine □Clinical medicine  Other research areas: | | | | | | |
| The type of supervisor you serve as: | □None □Master’s supervisor □ PhD supervisor | | | | | | |

**Part Two: Introduction to the Theory of** **Behaviour Change Wheel**

The Behaviour Change Wheel (BCW)describes eight steps for designing interventions that incorporate behavioral analysis using the COM-B model, aimed at understanding and exploring behaviors. Each component of behavior change corresponds to nine intervention functions (education, persuasion, incentivization, coercion, training, enablement, modeling, environmental restructuring, and restrictions) and seven policy strategies (environmental and social planning, communication and marketing, legislation, service provision, regulation, fiscal measures, and guidelines). The COM-B model acknowledges that behavior lies at the core of a complex interacting system, involving the capability (both physical and psychological), opportunity (social and physical), and motivation (both reflective and automatic) of individuals or groups to engage in specific behaviors.

**Third Part: Consultation Form for Experts**

Form Instructions: This section includes a total of 6 primary indicators, 16 secondary indicators, and 46 tertiary indicators for the construction of a virtual community exercise management program for Stage 2 cardiac rehabilitation post transcatheter aortic valve replacement patients. Please assess the importance of each indicator on a scale of 1 to 5, where 1 to 5 corresponds to 'Not important at all', 'Not important', 'Neutral', 'Important', and 'Very important' respectively. Please mark your assessment with a checkmark (√) under the appropriate option. If you believe some indicators are unnecessary or inaccurate, please provide your feedback in the 'Comments for Modification' section. If you feel additional indicators are needed, please add them in the 'Additional Indicators' section, and assess their importance similarly. Your profound expertise is crucial to the progress of this study. We sincerely appreciate your support and look forward to your valuable insights.

**The** **first-level indicators Indicator Assessment Form**

| First-level indicators | The importance of first-level indicators | | | | | Your editing suggestions |
| --- | --- | --- | --- | --- | --- | --- |
|  | 5  Very important | 4  Important | 3  Moderate | 2  Not important | 1  Not very important |  |
| 1. Enhancing the ability of patients with TAVR and their caregivers to manage risk factors. |  |  |  |  |  |  |
| 1. Enhancing the ability of patients with TAVR and their caregivers to manage anticoagulation at home. |  |  |  |  |  |  |
| 1. Enhancing the ability of patients with TAVR and their caregivers to manage complications and follow-up. |  |  |  |  |  |  |
| 1. Enhancing the ability of patients with TAVR to engage in home exercise. |  |  |  |  |  |  |
| 1. Creating an environment for home rehabilitation of patients with TAVR. |  |  |  |  |  |  |
| 1. Cultivating the motivation for home-based cardiac rehabilitation in patients with TAVR. |  |  |  |  |  |  |
| Supplementary indicators: |  |  |  |  |  |  |
| Your editing suggestions: | | | | | | |
| Note: New entries also need to be judged in terms of their level of importance. | | | | | | |

**Evaluation table for** **secondary-level indicators**

| First-level indicators | Secondary-level indicators | The importance of secondary-level indicators | | | | | Your editing suggestions |
| --- | --- | --- | --- | --- | --- | --- | --- |
|  |  | 5  Very important | 4  Important | 3  Moderate | 2  Not important | 1  Not very important |  |
| 1. Improve the risk management ability of TAVR patients and their caregivers. | 1.1. Providing patients with TAVR and their caregivers with knowledge on the management of cardiovascular disease risk factors. |  |  |  |  |  |  |
|  | 1.2. Guidance related to cardiovascular disease risk factor management skills for patients with TAVR and their caregivers. |  |  |  |  |  |  |
|  | - 1. Improvement in the management of cardiovascular disease risk factors in patients with TAVR and their caregivers. |  |  |  |  |  |  |
| 1. Improve the ability of TAVR patients and their caregivers to manage home anticoagulation. | - 1. Provide patients with TAVR and their caregivers with knowledge about anticoagulation management and home-based anticoagulation management. |  |  |  |  |  |  |
|  | - 1. Skills instruction in home anticoagulation self-monitoring for patients with TAVR and their caregivers. |  |  |  |  |  |  |
|  | - 1. Improving the safety of the anticoagulation of patients with TAVR. |  |  |  |  |  |  |
| 1. To enhance the complications and follow-up management capabilities of TAVR patients and their caregivers. | - 1. Provide patients with TAVR and their caregivers with knowledge about home-based complication follow-up management. |  |  |  |  |  |  |
|  | - 1. Improving the follow-up management skills of patients with TAVR and their caregivers. |  |  |  |  |  |  |
| 1. Improve the home exercise capacity of TAVR patients. | - 1. Provide patients with TAVR and their caregivers with knowledge about home-based exercises. |  |  |  |  |  |  |
|  | - 1. Risk providing skills instruction in-home exercise for patients with TAVR and Their Caregivers. |  |  |  |  |  |  |
|  | - 1. Improving the physical strength and endurance in patients with TAVR. |  |  |  |  |  |  |
| 1. Create a home-based rehabilitation environment for TAVR patients. | - 1. Formation of a multidisciplinary rehabilitation team to provide a medically supportive environment. |  |  |  |  |  |  |
|  | - 1. Re-establishing a home-based rehabilitation environment for patients with TAVR and providing opportunities for exercise. |  |  |  |  |  |  |
|  | - 1. Rebuilding the social environment of patients with TAVR and providing opportunities for rehabilitation. |  |  |  |  |  |  |
| 1. Motivating TAVR patients to engage in home-based cardiac rehabilitation. | - 1. Fostering reflective motivation for home-based rehabilitation in patients with TAVR. |  |  |  |  |  |  |
|  | - 1. Fostering spontaneous motivation for home-based cardiac rehabilitation in patients with TAVR. |  |  |  |  |  |  |
| Indicators that you suggest should be added: |  |  |  |  |  |  |  |
| Other recommendations: |  | | | | | | |

**Evaluation chart for the** **third****-level indicators**

| First-level indicators | Secondary-level Indicator | Third-level indicators | 5  Very important | 4  Important | 3  Moderate | 2  Not important | 1  Not very important | 5  Very important |
| --- | --- | --- | --- | --- | --- | --- | --- | --- |
| 1. Improve the cardiovascular risk factor management capabilities of patients with TAVR and their caregivers. | - 1. Providing relevant knowledge on cardiovascular disease risk factor management for patients with TAVR and their caregivers. | - - 1. Rehabilitation teams use WeChat mini-programs to deliver personalized risk assessment videos to patients. These videos include assessments of cardiac rehabilitation risks, such as medical history, cardiac functional indicators, valve function, frailty, nutritional status, sleep quality, related complications and comorbidities, flexibility, and balance function, daily life activities, psychological state, lifestyle, socioeconomic factors, digital health literacy, usage of wearable devices and smartphones, acceptance of mobile devices, and information needs related to diseases. Assessments are dynamically personalized based on the patient's mental and physical condition, emphasizing timing, purpose, and significance. |  |  |  |  |  |  |
|  |  |  |  |  |  |  |  |  |
|  |  |  |  |  |  |  |  |  |
|  |  | - - 1. The nurse uses WeChat mini-programs to deliver educational videos on cardiovascular risk health according to the rehabilitation plan. The content includes information on valvular heart disease, blood pressure, lipid levels, mental health, sleep management, smoking cessation, and nutrition management. |  |  |  |  |  |  |
|  |  | - - 1. The nurse provided videos on the use of WeChat mini-programs and mobile devices, covering the purposes, as well as the purposes, methods, and precautions of wearable devices. |  |  |  |  |  |  |
|  | - 1. Guide cardiovascular disease risk factor management skills for patients with TAVR and their caregivers. | - - 1. Nurses teach patients to use WeChat mini-programs (such as online consultations, nutrition diaries, and dietary records) and wearable devices through on-site face-to-face teaching and remote video teaching within the hospital. |  |  |  |  |  |  |
|  |  | - - 1. Nurses teach patients self-monitoring techniques, as well as how to identify and manage dangerous symptoms through face-to-face teaching within the hospital and remote video instruction. |  |  |  |  |  |  |
|  | - 1. Enhancing the ability of patients with TAVR and their caregivers to manage cardiovascular risk factors. | - - 1. The rehabilitation team sets personalized management goals for risk factors such as blood pressure, blood lipids, blood glucose, smoking cessation, alcohol restriction, and weight based on the patient's condition. |  |  |  |  |  |  |
|  |  | - - 1. The rehabilitation team uses methods such as cognitive behavioral therapy, motivational interviewing, case management, stress reduction through mindfulness meditation, yoga, relaxation sessions, video conferences, etc., to intervene in cardiovascular disease risk factors. |  |  |  |  |  |  |
|  |  | - - 1. Encourage patients to use WeChat mini-programs to upload pictures or text, record waist circumference, sleep, weight, calorie consumption, and other data, and regularly remind patients to use medication adherence questionnaires, self-care assessment scales, and other patient self-report outcome measures to improve patient compliance. |  |  |  |  |  |  |
|  |  | - - 1. The rehabilitation team develops personalized rehabilitation plans based on the patient’s values and preferences, regularly assesses the patient’s exercise, sleep, nutrition, and management of risk factors using data from the WeChat mini-program, and provides feedback to the patient. |  |  |  |  |  |  |
| 1. To enhance the ability of TAVR patients and their caregivers to manage anticoagulation therapy at home. | - 1. Providing home anticoagulation management knowledge for patients with TAVR and their caregivers. | - - 1. The rehabilitation team uses the WeChat Mini Program to push videos on postoperative anticoagulant medication. The content includes usage methods of commonly used drugs, precautions for antithrombotic and anticoagulant drug use, drug interactions, medication complications, and more. |  |  |  |  |  |  |
|  | - 1. Providing home anticoagulation self-monitoring skills guidance for patients with TAVR and their caregivers. | - - 1. The nurse teaches patients the correct sampling techniques through face-to-face guidance combined with assistance from mini-program videos and instructs patients on the proper use of portable coagulation meters. |  |  |  |  |  |  |
|  | - 1. Improving the Safety of Anticoagulation Therapy in Patients with TAVR. | - - 1. Rehabilitation teams use WeChat mini-programs to communicate dynamically with patients. They dynamically assess the bleeding risk and thrombosis of patients after medication. |  |  |  |  |  |  |
| 1. To enhance the home complications and follow-up management capabilities of TAVR patients and caregivers. | - 1. Providing relevant knowledge on home-based complication follow-up management for patients with TAVR and their caregivers. | - - 1. The nurses regularly send videos through WeChat mini-programs to educate patients about the prevention, symptoms, and management of postoperative long-term complications. They also teach patients to recognize symptoms such as unusual chest pain, back pain or discomfort, significant palpitations or irregular heartbeats, limb swelling or rapid weight gain, fever, chills, and persistent nausea. |  |  |  |  |  |  |
|  |  | - - 1. The rehabilitation team uses WeChat mini-programs and wearable devices to dynamically track patients' management of complications, regularly pushing content on self-observation and prevention of relevant complications to patients. |  |  |  |  |  |  |
|  |  | - - 1. The nurse delivers home follow-up through WeChat mini-programs to achieve the following purposes: identification of clinical status changes, management of antithrombotic therapy, monitoring complications, recognition of valve function changes, and provision of patient education. Time points include follow-ups at 1, 3, 6, and 12 months post-discharge, adjusting rehabilitation guidance based on results. After one year, if no significant discomfort occurs, annual follow-up reviews are recommended. The focus is on evaluating valve function, peri-valvular leaks, endocarditis, long-term durability of valves, transvalvular gradients, regurgitation, stenosis, leaflet calcification or thrombus formation, assessing signs of infection, conduction abnormalities, or myocardial infarction, managing complications, and adjusting medications and rehabilitation plans as necessary. |  |  |  |  |  |  |
|  |  | - - 1. Nurses use WeChat mini-programs to push follow-up examination content. |  |  |  |  |  |  |
|  |  | - - 1. Nurses use WeChat mini-programs to push follow-up care instructions to patients. |  |  |  |  |  |  |
|  |  | - - 1. The nurse introduces rehabilitation outcome indicators to patients through the WeChat mini-program, including quality of life, nutritional status, and incidence of complications. |  |  |  |  |  |  |
|  | - 1. Enhancing follow-up management capabilities for patients with TAVR and their caregivers. | - - 1. The rehabilitation team uses WeChat Mini Program message reminders to send messages to patients at scheduled times every day, reminding them regularly to carry out rehabilitation measures and conduct follow-ups. |  |  |  |  |  |  |
|  |  | - - 1. Rehabilitation teams need to help patients identify and overcome barriers to health-related behaviors through face-to-face and remote communications. For patients who live alone, have low educational levels, or suffer from depression, the rehabilitation team should provide professional assistance. |  |  |  |  |  |  |
|  |  | - - 1. The rehabilitation team combines outpatient follow-ups with WeChat mini-program follow-ups to promptly learn about adverse events and the self-management of cardiac rehabilitation from patients. They maintain continuous communication with patients, overseeing the execution of their home rehabilitation plans. |  |  |  |  |  |  |
| 1. Improving home exercise capacity for TAVR patients. | - 1. Provide relevant knowledge on home exercises for patients with TAVR and their caregivers. | - - 1. Nurses use WeChat mini-programs to send home exercise videos to patients. The content includes types of home exercises, the importance of exercise, intensity, frequency, and duration of home exercise rehabilitation, possible adverse reactions during exercise and corresponding measures, signs to stop exercising, and special considerations for specific groups of people. |  |  |  |  |  |  |
|  |  | - - 1. The rehabilitation team uses WeChat mini-programs to push commonly used indicators for exercise monitoring, including Physical performance indicators, Psychological indicators such as anxiety levels, depression levels, quality of life scores, and Biological indicators. |  |  |  |  |  |  |
|  | - 1. Providing home exercise skill guidance for patients with TAVR and their caregivers. | - - 1. The rehabilitation team provides patients with videos on home aerobic endurance exercises, gravity-resisting exercises, standing balance and coordination training, resistance training exercises, and traditional Chinese medicine rehabilitation exercises through inpatient face-to-face guidance and remote video teaching. |  |  |  |  |  |  |
|  |  | - - 1. Nurses use WeChat mini-programs to push methods for using pulse oximeters and fatigue level tests, teaching patients home exercise recovery techniques and self-assessment skills, enabling patients to recognize dangerous symptoms and rescue themselves. |  |  |  |  |  |  |
|  | - 1. Improving physical fitness and endurance in patients with TAVR. | - - 1. Encourage patients to use pedometers, portable ECG monitors, health diaries, etc., to dynamically monitor their exercise at home. Playing music with a consistent rhythm during exercise can enhance interest in physical activities. |  |  |  |  |  |  |
|  |  |  |  |  |  |  |  |  |
|  |  | - - 1. The rehabilitation team can use meetings, motivational text messages, phone calls, WeChat Mini Program notifications, etc., to understand the patients' exercise situations and feelings. They can track the patients' exercise status using WeChat Mini Programs and wearable devices, and adjust exercise plans promptly. |  |  |  |  |  |  |
| 1. Creating a home recovery environment for TAVR patients. | - 1. Establish multidisciplinary rehabilitation teams to provide a supportive medical environment. | - - 1. To form a multidisciplinary rehabilitation team to screen patients participating in home-based rehabilitation, and to develop an emergency plan for home cardiac rehabilitation. |  |  |  |  |  |  |
|  |  | - - 1. The rehabilitation team is responsible for producing educational manuals and promotional videos on cardiac rehabilitation. All promotional content is reviewed and proofread by relevant medical experts, specifying the sources of information, publication dates, and references. The names and affiliations of experts involved in the review, their contributions, and their respective units are listed at the end of the manual. The promotional manual should avoid using professional terminology and use language that is clear, concise, and easy to understand. |  |  |  |  |  |  |
|  |  | - - 1. The health-related information provided by the team on the WeChat mini-program should be action-oriented, including clear and concise information with clear language articulation. When the rehabilitation team carries out remote cardiac rehabilitation interventions, they should promptly record the corresponding medical/nursing records. |  |  |  |  |  |  |
|  |  | - - 1. The health-related information about specific treatments, products, services, or behaviors provided on WeChat mini-programs should clearly outline the associated risks and benefits. |  |  |  |  |  |  |
|  |  | - - 1. Health-related information on WeChat Mini Programs should respect principles of privacy and confidentiality. For example, if sharing information, images, or videos involving others or about others, permission should be obtained before sharing. |  |  |  |  |  |  |
|  | - 1. Reconstructing home rehabilitation environments for patients with TAVR, providing exercise opportunities. | - - 1. Before discharge, the rehabilitation team provides patients with wearable devices, smartphones, blood pressure monitors, fingertip pulse oximeters, activity trackers, mobile electrocardiograms, and chest-worn heart rate monitors or heart rate monitors to dynamically monitor exercise indicators and ensure exercise safety. |  |  |  |  |  |  |
|  |  |  |  |  |  |  |  |  |
|  |  |  |  |  |  |  |  |  |
|  |  |  |  |  |  |  |  |  |
|  |  | - - 1. The rehabilitation team established a closed-home cardiac rehabilitation management system that combines a patient-side remote monitoring system, smart wearable devices, mobile applications, and a medical-side website system. This system dynamically monitors the recovery status of patients. |  |  |  |  |  |  |
|  | - 1. Rebuild the social environment of patients with TAVR and provide rehabilitation opportunities. | - - 1. The community health service center needs to establish a comprehensive cardiac rehabilitation center with assessment, treatment, and follow-up capabilities. The spatial design should include areas for emergency care, assessment, exercise, and health education. It should be equipped with aerobic exercise equipment such as stationary bikes and treadmills, as well as resistance training equipment for the upper and lower limbs. Additionally, it should have devices like percutaneous blood oxygen saturation monitors, blood pressure monitors, blood glucose monitors, oxygen inhalers, electrocardiographs, telemetry exercise ECG monitoring systems, automated external defibrillators, and emergency medications in a rescue vehicle, with at least one cardiopulmonary assessment testing system. This setup facilitates organized group rehabilitation exercise programs and ensures emergency rescue during critical patient exercise moments. |  |  |  |  |  |  |
|  |  | - - 1. Encourage patients to join rehabilitation groups, participate in online/offline rehabilitation communities, and share experiences and achievements with other patients to increase interaction and motivation. |  |  |  |  |  |  |
|  |  | - - 1. The rehabilitation team regularly holds online health knowledge tests, online cardiac rehabilitation workshops, online group discussions, and other activities to promote home-based rehabilitation. |  |  |  |  |  |  |
|  |  | - - 1. Caregivers can provide comprehensive support and assistance to patients through emotional support, rehabilitation assistance, dietary management, companionship and communication, rehabilitation monitoring, and improving the home environment, reminding patients to exercise regularly. |  |  |  |  |  |  |
| 1. Motivating home cardiac rehabilitation for TAVR patients. | - 1. Cultivating reflective motivation for home-based rehabilitation in patients with TAVR. | - - 1. The rehabilitation team uses WeChat mini-programs to promote the concept and importance of home rehabilitation, emphasizing the benefits of cardiac rehabilitation. They remind individuals to consider the possible health outcomes of adhering to or not adhering to exercise-based rehabilitation, aiming to stimulate patients’ reflective motivation. |  |  |  |  |  |  |
|  |  | - - 1. The rehabilitation team collaborates with patients online to develop rehabilitation plans together. They use an application to record patients’ adherence to exercises, stimulate patients’ execution of functional exercises, evaluate patients’ compliance with exercise routines, provide feedback, and continuously improve. |  |  |  |  |  |  |
|  |  | - - 1. The rehabilitation team pushes the hazards of heart valve disease through WeChat mini-program, establishes patients' susceptibility to adverse outcomes such as reduced heart function, heart failure, arrhythmia, etc., introduces the incidence and influencing factors of various adverse events during postoperative long-term complications, and the hazards of not conducting cardiac rehabilitation after surgery, making patients aware of the seriousness of adverse outcomes such as endocarditis infection, stroke, myocardial infarction, weakness, etc. |  |  |  |  |  |  |
|  |  | - - 1. The rehabilitation team can provide patients with TAVR postoperative success and poor recovery cases, pictures, and videos as needed, based on the patient's condition, to make patients aware of the importance of rehabilitation exercises. |  |  |  |  |  |  |
|  | - 1. Cultivating spontaneous motivation for home-based cardiac rehabilitation in patients with TAVR. | - - 1. The rehabilitation team praises patients appropriately based on their recovery progress at each stage (after completing tasks, they can unlock new tools or skills, earn virtual currency or medals, and achieve titles on the leaderboard), providing positive feedback to enhance their motivation for exercise. |  |  |  |  |  |  |
|  |  |  |  |  |  |  |  |  |
|  |  | - - 1. Rehabilitation teams share successful rehabilitation cases, with compliant patients sharing personal rehabilitation experiences to instill confidence in overcoming difficulties for other patients. |  |  |  |  |  |  |
|  |  | - - 1. Rehabilitation teams regularly select the most outstanding patients and caregivers, awarding them electronic certificates to enhance their motivation and engagement in the process. |  |  |  |  |  |  |

**Part IV: Expert Authority Survey**

Judgmental basis refers to the basis of the importance score you give to the entry, please judge "theoretical basis", "practical experience", "domestic and foreign literature", Please judge the degree of influence of the four aspects of "theoretical basis", "practical experience", "domestic and foreign literature" and "subjective intuition" on the score you have given, and put a tick in the corresponding column. At the same time, please judge your familiarity with the content of the study and tick in the appropriate column.

| **Your basis for judging the content of this study (expert self-assessment)** | | | |
| --- | --- | --- | --- |
| Basis for content judgment | Degree of impact | | |
|  | High | Moderate | Low |
| Theoretical foundation |  |  |  |
| Practical/scientific experience |  |  |  |
| Documentation |  |  |  |
| Subjective instinct |  |  |  |

| **Your familiarity with the content of this study** | | | | | |
| --- | --- | --- | --- | --- | --- |
| Content familiarity | Very familiar | Comparatively familiar | Fairly familiar | Not too familiar | Not familiar |
| Expert self-assessment |  |  |  |  |  |
